# The Germ Cell Determinant Blimp1 Is Not Required for Derivation of Pluripotent Stem Cells

**DOI:** 10.1016/j.stem.2012.02.023

**Published:** 2012-07-06

**Authors:** Siqin Bao, Harry G. Leitch, Astrid Gillich, Jennifer Nichols, Fuchou Tang, Shinseog Kim, Caroline Lee, Thomas Zwaka, Xihe Li, M. Azim Surani

**Affiliations:** 1 Wellcome Trust/Cancer Research UK Gurdon Institute of Cancer and Developmental Biology, University of Cambridge, Tennis Court Road, Cambridge, CB2 1QN, UK; 2 Department of Physiology, Development, and Neuroscience, University of Cambridge, Tennis Court Road, Cambridge, CB2 1QN, UK; 3 Center for Cell and Gene Therapy, and Departments of Molecular and Cellular Biology and Human Genetics, Baylor College of Medicine, Houston, TX 77030, USA; 4 College of Life Science, Inner Mongolia University/Mengniu RB CO. Ltd., No. 235 Da Xue Xi Road, Huhhot, Inner Mongolia 010021, China

## Abstract

Blimp1 (Prdm1), the key determinant of primordial germ cells (PGCs), plays a combinatorial role with Prdm14 during PGC specification from postimplantation epiblast cells. They together initiate epigenetic reprogramming in early germ cells toward an underlying pluripotent state, which is equivalent to embryonic stem cells (ESCs). Whereas Prdm14 alone can promote reprogramming and is important for the propagation of the pluripotent state, it is not known whether Blimp1 is similarly involved. By using a genetic approach, we demonstrate that Blimp1 is dispensable for the derivation and maintenance of ESCs and postimplantation epiblast stem cells (epiSCs). Notably, Blimp1 is also dispensable for reprogramming epiSCs to ESCs. Thus, although Blimp1 is obligatory for PGC specification, it is not required for the reversion of epiSCs to ESCs and for their maintenance thereafter. This study suggests that reprogramming, including that of somatic cells to ESCs, may not entail an obligatory route through a Blimp1-positive PGC-like state.

## Graphical abstract

**Graphical Abstract:**

**Figure:**
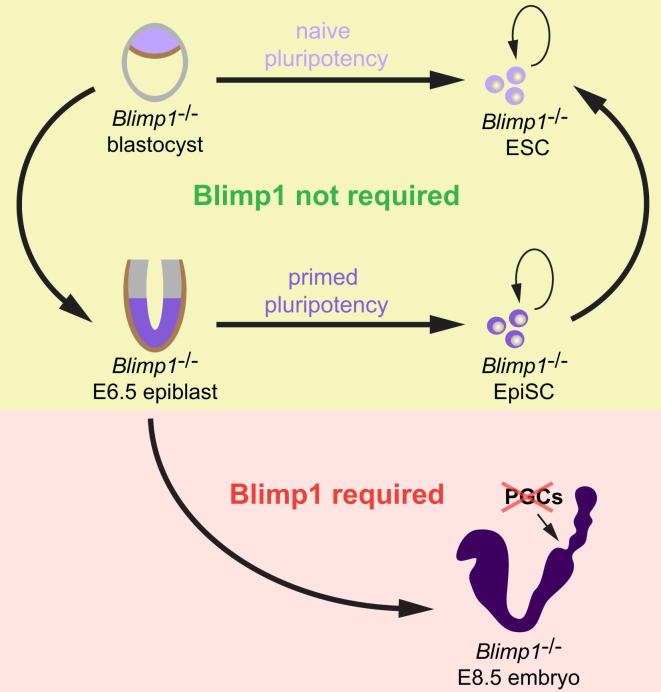

**Highlights:**

► Knockout of Blimp1 has no effect on derivation or maintenance of ESCs or epiSCs ► Blimp1 is not required for reversion of epiSCs to an ESC state ► Blimp1 is required for PGC specification ► Transit through a PGC state is not obligatory for acquisition of pluripotency

## Teaser

Bao et al. demonstrate that the key determinant of primordial germ cells (PGCs) Blimp1 is dispensable for the derivation of embryonic stem cells (ESCs) and epiblast stem cells (epiSCs) and for reprogramming. This suggests that acquisition of pluripotency does not entail an obligatory route through a Blimp1-positive PGC-like state.

## Introduction

Expression of Blimp1, the key regulator of PGC specification, is obligatory for the establishment of the germ cell lineage in mice (Ohinata et al., 2005; Vincent et al., 2005). Blimp1 expression is first detected in a few proximal postimplantation epiblast cells at embryonic day (E) 6.25, which results in 30–40 founder PGCs at E7.5 (Ohinata et al., 2005; Vincent et al., 2005). Blimp1 together with Prdm14 plays a critical role in early germ cells as they induce repression of the somatic program, initiation of PGC program-coupled epigenetic reprogramming, and re-expression of pluripotency genes (Ohinata et al., 2005; Yamaji et al., 2008). Thus, although PGCs are unipotent, they have an epigenetic state and other properties, such as active X chromosomes in female PGCs, which resemble key features of the inner cell mass (ICM) of blastocysts and ESCs. The reversion and reprogramming of postimplantation epiblasts and epiSCs to reverted ESC-like cells (henceforth called rESCs) is accompanied by similar epigenetic changes to those seen during PGC specification and early germ cells (Hajkova et al., 2008; Surani et al., 2007; Bao et al., 2009).

Recent studies have shown that Prdm14 has a role in the maintenance of mouse ESCs partly through the repression of differentiation (Ma et al., 2011), and it is also obligatory for the persistence of pluripotency in human ESCs (Chia et al., 2010). Furthermore, Prdm14 enhances epigenetic reprogramming of human and mouse somatic cells to induced pluripotent stem cells (iPSCs) (Chia et al., 2010; Nagamatsu et al., 2011). Prdm14 acts in conjunction with Blimp1 to induce epigenetic reprogramming in PGCs and early germ cells (Yamaji et al., 2008), suggesting that they play a combinatorial role in the germ cell lineage. This, together with other observations, has led to a notion that reprogramming in other contexts, including that of somatic cells to a ground state of pluripotency seen in ESCs, might entail a transition through a PGC-like state (Zwaka and Thomson, 2005; Nichols and Smith, 2011; Nagamatsu et al., 2011; Chu et al., 2011).

EpiSCs, which are derived from postimplantation epiblast cells, inherit some of the key properties from them, including an inactive X chromosome in female cells, which differ in many other respects too, including their epigenetic state compared to the ESCs derived from the ICM of blastocysts (Tesar et al., 2007; Brons et al., 2007). Furthermore, epiSCs gain additional DNA methylation at some loci, such as stella (*Dppa3*) and Rex1, during their derivation from epiblast cells (Bao et al., 2009). We showed previously that some epiSCs can undergo PGC specification after expression of Blimp1 and Prdm14 accompanied by appropriate epigenetic reprogramming, consistent with observations on PGCs in vivo (Hayashi and Surani, 2009). Furthermore, the reversion of epiSCs to rESCs in response to leukaemia inhibitory factor (LIF)-Stat3 is similarly accompanied by epigenetic reprogramming, X reactivation, re-expression of pluripotency genes, DNA demethylation, and repression of somatic genes (Bao et al., 2009; Hanna et al., 2009; Yang et al., 2010; Greber et al., 2010). Thus, there are some key shared features of epigenetic reprogramming of epiSCs during PGC specification and during reversion to rESCs.

We previously excluded a possibility that rESCs may be derived from dedifferentiating PGCs (Bao et al., 2009). However, further evidence is required to exclude this likelihood unequivocally, and particularly also a possibility that the reversion of epiSCs to rESCs could involve a transition through a PGC-like state, especially as they share some key features of epigenetic reprogramming. If so, we would anticipate a key role for Blimp1 during the reversion of epiSCs to rESCs and possibly in other instances of reprogramming of somatic cells to iPSCs in vitro. Note that Blimp1 is at the same time also essential for differentiation of some somatic cells later during embryogenesis (Robertson et al., 2007).

In this study we investigated whether Blimp1 is required for the generation and maintenance of the ESC state. Our study shows that whereas Blimp1 is obligatory for PGC specification, it is dispensable during the derivation of ESCs and epiSCs, as well as during the reversion of epiSCs to ESCs and their long-term maintenance thereafter as self-renewing pluripotent stem cells. *Blimp1*^−/−^ ESCs are also capable of differentiating into somatic cells in chimeras, and development as early postimplantation embryos in tetraploid rescue experiments, but they cannot give rise to the germ cell lineage.

## Results and Discussion

### Assessment of the Requirement of Blimp1 for ESC Derivation

**Figure 1:**
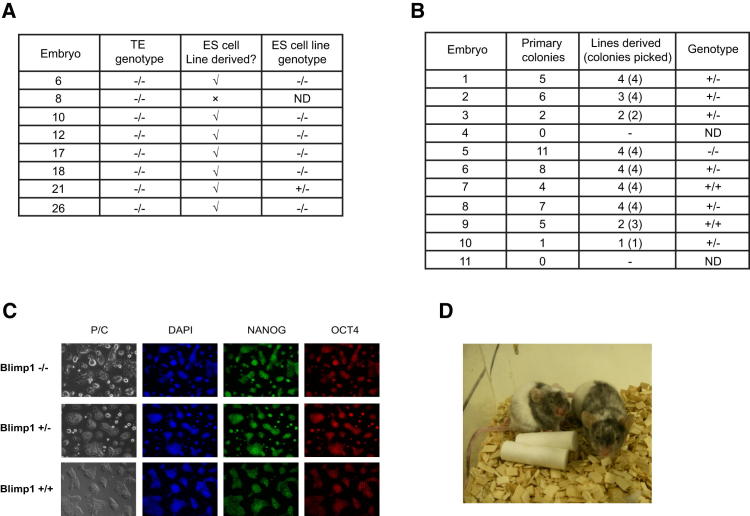
Derivation of *Blimp1*^−/−^ ESCs from Blastocysts (A) Summary of *Blimp1*^−/−^ ESC derivations from whole ICMs. (B) Summary of ESC derivations from trypsinized ICMs plated as single cells. (C) Oct4 and Nanog immunostaining of *Blimp1*^−/−^, *Blimp1*^+/−^, and *Blimp1*^+/+^ ESCs. (D) Chimeras generated with *Blimp1*^−/−^ ESCs (dark agouti) injected into albino C57BL/6 blastocysts. See also Tables S1 and S2.

First, we set out to test whether Blimp1 is essential for the establishment of ESCs. To do so, we intercrossed mice heterozygous for a Blimp1 mutant allele (Ohinata et al., 2005) and retrieved 8-cell stage embryos. These embryos were cultured in medium supplemented with the small molecules PD0325901 and Chir99021 (2i) to inhibit the protein kinase (Erk1/2) cascade and glycogen synthase kinase, respectively (Nichols et al., 2009; Ying et al., 2008). After 3 days, all embryos formed expanded blastocysts and hatched from the zona pellucida. The ICMs were isolated from expanded blastocysts by immunosurgery and transferred to 2i medium supplemented with LIF. The outer trophectoderm cells from individual embryos were retained and used to genotype the respective epiblast. We found 8/40 embryos to be null for Blimp1 by trophectoderm genotyping (Figure 1A). These epiblasts were allowed to grow for a further 4 days and primary colonies were expanded as ESC lines. Lines were established from 7/8 embryos and each line was regenotyped, which confirmed that 6/7 ESC lines were null for Blimp1 (Figure 1A). In a separate experiment, an ESC line was established from each of 10 embryos, and one of these was shown to be null for Blimp1 (Table S1 available online). These experiments show that it is possible to derive ESC lines directly from *Blimp1*^−/−^ blastocysts. Note that the 2i conditions are not essential for the establishment of ESCs as shown by the fact that reversion of epiSCs to rESCs occurred efficiently under classical culture conditions with LIF and fetal calf serum (see later).

To test whether the Blimp1-null ESCs are in any way compromised, we tested their colony-forming ability at the single cell level. In this case, we obtained 11 embryos at E4.5 by crossing *Blimp1*^+/−^ heterozygous mice and repeated the procedure for ESC derivation described above, except that the ICM from each embryo was dissociated into single cells and then dispersed onto a feeder layer in a 48-well culture dish. The colonies in each of the wells were counted after 5 days. Two embryos, which had small ICMs, produced no colonies. Cells from the remaining 9 ICMs produced between 1 and 11 colonies (Figure 1B), consistent with previous findings (Nichols et al., 2009). Individual colonies were picked and multiple ESC lines were established from each embryo, with the exception of embryo 10, which produced only one primary colony. Each ESC line was genotyped for Blimp1 and in every case lines derived from the same embryo were of the same genotype (data not shown). For example, all 4 ESC lines from embryo 5, which produced 11 primary colonies, were Blimp1 null (Figure 1B), which is comparable with the maximum efficiency reported previously (Nichols et al., 2009). This demonstrates that the derivation of ESCs from Blimp1 mutant embryos occurs efficiently and is not detectably compromised. Indeed, from 3 independent ESC derivation experiments, we have obtained a total of 11 separate Blimp1-null ESC lines from 9 Blimp1-null blastocysts. Blimp1-null ESCs, as well as control heterozygous and wild-type lines, were immunoreactive for the key pluripotency transcription factors Oct4 and Nanog (Figure 1C).

Next, we carried out a functional test on *Blimp1*^−/−^ ESCs by examining whether they can participate in forming chimeric adult mice by injecting them into wild-type host blastocysts. We observed extensive contribution of *Blimp1*^−/−^ ESCs as judged by their contribution to coat color. However, as expected, we did not observe germline transmission in the absence of Blimp1 (Figure 1D; Table S2). We had similar results with rESCs (see later). We conclude that pluripotent ESCs can be established efficiently from embryos with homozygous genetic deletion of Blimp1. We do not rule out that ESC derivation by alternative strategies or from particular mouse strains may require Blimp1 activity. The ESC lines established here were of a mixed, predominantly C57BL/6 and CBA, genetic background; Blimp1-null ESCs of the permissive 129 homozygous genetic background are phenotypically similar to that of the mixed genetic background (data not shown). All these ESC lines are indistinguishable from wild-type ESCs in culture. We have maintained *Blimp1*^−/−^ ESCs for more than 30 passages both in 2i/LIF conditions as well as in conventional cultures with fetal calf serum (FCS) and LIF (with or without feeders) for more than 15 passages without detectable effects on the properties of these ESCs.

### Derivation of EpiSCs from *Blimp1*^−/−^ Postimplantation Epiblast Cells

We next asked whether it is possible to derive epiSC lines from *Blimp1*^−/−^ postimplantation epiblast cells. For this purpose, we used established *Blimp1*^−/−^ ESC lines (129 background) and injected them into wild-type tetraploid host blastocysts, which contribute almost exclusively to extraembryonic tissues, including the visceral endoderm, while the donor ESCs contribute to the embryo proper (Nagy et al., 2003).

**Figure 2:**
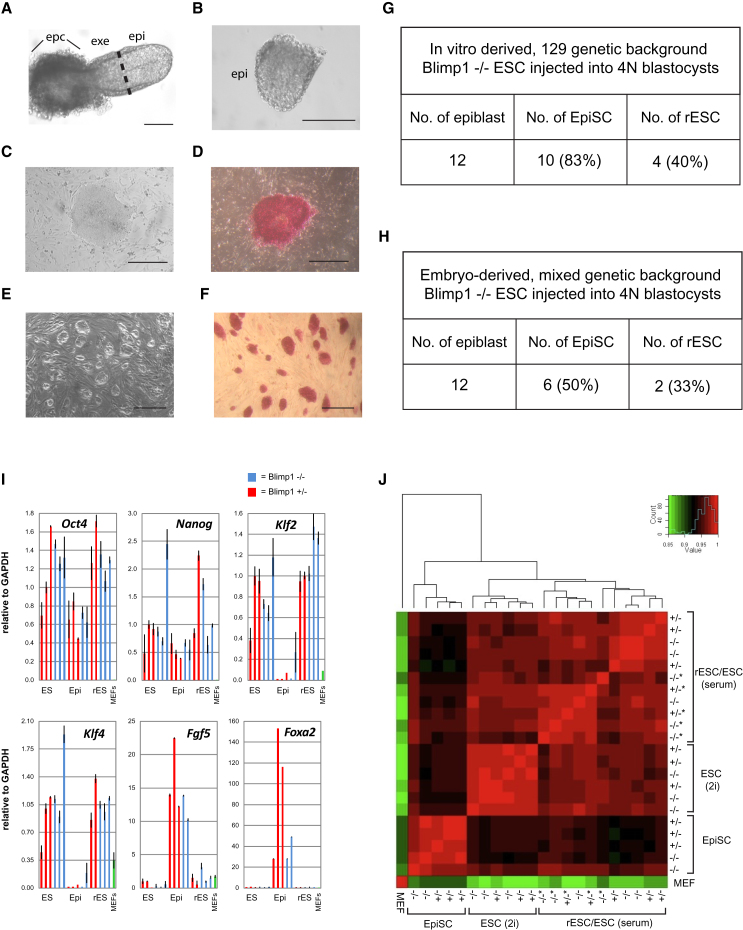
Reprogramming of *Blimp1*^−/−^ EpiSCs to rESCs and Expression Analysis of Blimp1-null Pluripotent Stem Cell Lines (A) Embryo at E6.5 generated from *Blimp1*^−/−^ ESCs after injection into 4N teraploid host blastocyst. Epiblast tissue was divested of the proximal region (black line). epi, epiblast; exe, extraembryonic ectoderm; epc, ectoplacental cone. (B) Dissected epiblast tissue. (C) Derivation of epiSCs from epiblast. (D) AP staining in epiSCs. (E) Derivation of rESCs from epiSCs. (F) Uniform AP staining of rESCs. Scale bars represent 200 μm. (G and H) Number of *Blimp1*^−/−^ rESCs from *Blimp1*^−/−^ epiSCs of 129 inbred genetic background (G) and mixed genetic background (H). (I) qRT-PCR analysis of *Blimp1*^−/−^ epiSCs, ESCs, and rESCs. rESCs and ESCs were cultured in FCS/LIF. Heterozygous cell lines and mouse embryonic fibroblasts (MEFs) were used as control. At least two, and usually three, biological replicates were analyzed for each cell type and genotype. Error bars denote the standard deviation of two technical replicates. (J) Correlation heatmap generated after microarray analysis of the cell lines analyzed in (I). The same *Blimp1*^−/−^ and *Blimp1*^+/−^ ESCs cultured in 2i/LIF were also included. Asterisk denotes rESC lines. See also Figure S1 and Table S3.

Twelve embryos were isolated at E6.5 for the derivation of epiSCs (Figure 2A). The epiblast tissue was dissected to remove the most proximal region and the outer visceral endoderm (Figure 2B). The resulting cells were cultured in Activin A and bFGF in a chemically defined serum replacement medium (henceforth called CDM) on mouse embryonic fibroblasts feeders (MEFs) as described previously (Bao et al., 2009). After 4–7 days, we detected large colonies in 10/12 cultures with many alkaline phosphatase (AP)-positive cells (Figures 2C and 2D). We propagated these epiSC colonies in CDM by collagenase treatment without detectable morphological changes for at least 20 passages. Notably, we obtained similar epiSC lines both from the inbred 129 and mixed genetic background. Both wild-type and Blimp1-null epiSCs showed a similar morphology and could be maintained in culture thereafter (Figures 2G and 2H).

### Investigation of the Requirement of Blimp1 for Reversion of EpiSCs to rESCs

After establishment of 10 *Blimp1*^−/−^ epiSC lines, we tested their ability to undergo reversion to rESCs by transferring them to medium containing LIF and FCS as described previously (Bao et al., 2009). After 12–30 days in culture, we started to detect clusters of cells with a different morphology from the original epiSCs. Subsequent culture of these cells was carried out after disruption of the developing colonies by treatment with trypsin, which is detrimental to the remaining epiSCs but promotes propagation of ESC-like cells. With further passaging, we established multiple Blimp1-null rESC lines (4/10) (Figures 2E and 2F). We also derived *Blimp1*^−/−^ epiSCs from *Blimp1*^+/−^ heterozygous intercrosses (Figures S1A and S1B). These too readily reverted to give rESC lines, and notably the dynamics of reprogramming was indistinguishable when compared with reversion of epiSCs derived from Blimp1 heterozygous littermate (Figure S1C).

Next we analyzed the gene expression profile of *Blimp1*^−/−^ ESCs, epiSCs, rESCs, and heterozygous control lines by quantitative RT-PCR (qRT-PCR). All lines expressed the pluripotency factors Oct4 and Nanog (Figure 2I). EpiSC lines expressed low levels of Klf2 and Klf4 and high levels of Fgf5 and Foxa2 (Figure 2I). In contrast, rESCs displayed a gene expression pattern indistinguishable from ESCs, indicating successful reprogramming. There was no obvious effect of loss of Blimp1 on the gene expression profile of epiSCs, ESCs, or rESCs (Figure 2I). Female Blimp1-null epiSCs also exhibited nuclear H3K27me3 foci, which is lost upon reversion to rESCs, consistent with the reactivation of the inactive X chromosome that occurs as efficiently in the absence of Blimp1 (Figure S1D).

Next we investigated the transcriptome of Blimp1-null pluripotent stem cells by microarray analysis. The rESC and ESC lines clustered together, indicating successful transcriptional reprogramming during the reversion process and were clearly distinct from epiSCs (Figure 2J). Direct comparison between rESC and epiSC lines showed 3,868 differentially expressed genes (false discovery rate [FDR] adjusted p value < 0.01). ESCs cultured in 2i/LIF also formed a discrete cluster, suggesting a broad transcriptional change in this condition, consistent with observations in our laboratory (H.G.L. and M.A.S., unpublished observations). However, there was no detectable effect on cells with a loss of Blimp1 in any of the cell types we tested. Pairwise comparisons between epiSCs showed a correlation of >0.96 between cells with and without Blimp1, with fewer than 400 genes differentially expressed in any single comparison (Figure S1E). Such small variations are routinely evident even between heterozygous epiSC lines, which are consistent with published data for wild-type epiSCs (Figure S1E; Han et al., 2011). Furthermore, comparisons between Blimp1-null ESCs (or rESCs) and control lines revealed no differentially expressed genes, in either standard or 2i/LIF culture conditions (FDR adjusted p value < 0.01 for each comparison). These results indicate that Blimp1-null pluripotent stem cells at this stage are highly similar if not identical with normal ESCs, notwithstanding their inability to contribute to the germline and some somatic tissues later in embryogenesis.

**Figure 3:**
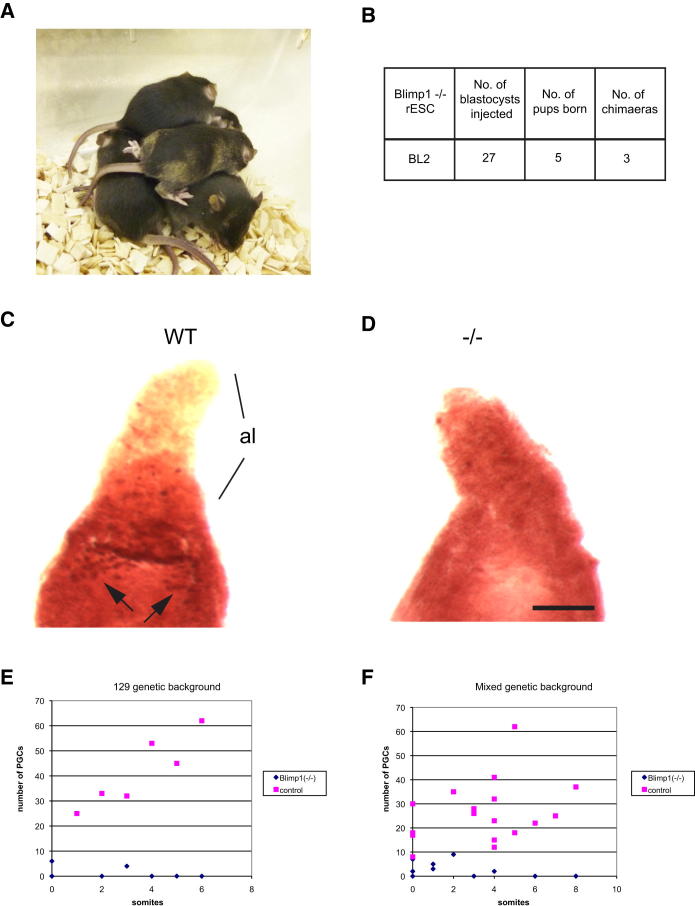
PGCs in Embryos Generated from *Blimp1*^−/−^ rESCs and control rESCs (A) Chimeras generated with *Blimp1*^−/−^ rESCs (dark agouti) injected into C57BL/6 (black) blastocysts. (B) Summary of blastocyst injections. (C and D) Comparison of PGCs detected by AP staining at E8.5 in WT control (C) embryos, versus *Blimp1*^−/−^ (D) embryos, revealed migrating PGCs in wild-type embryos (arrowheads) and only a few nonmigrating AP-positive cells in *Blimp1*^−/−^ embryos. al, allantois. Scale bar represents 200 μm. (E) Number of PGCs detected in control E8.5 embryos (n = 6) and *Blimp1*^−/−^ E8.5 embryos (n = 8) of 129 genetic background *Blimp1*^−/−^ rESCs. (F) PGCs from control E8.5 embryos (n = 17) and mutant rESC-derived E8.5 embryos (n = 12) of mixed genetic background. See also Figure S2.

Blimp1-null rESCs, like Blimp1-null ESCs, can contribute to chimeras (Figures 3A and 3B), which provides functional proof for complete reversion in the absence of Blimp1. Next we checked whether *Blimp1*^−/−^ rESCs contribute to the entire developing embryo by using the “tetraploid rescue” experimental approach (Nagy et al., 2003). We obtained comparable E8.5 embryos from both normal and *Blimp1*^−/−^ rESCs (Figures S2A and S2B), indicating their potential for extensive differentiation. Note that Blimp1 is important later for development of some somatic cells and this will influence differentiation of *Blimp1*^−/−^ cells in some tissues in chimeras (Robertson et al., 2007), which is in contrast to its role under consideration in this investigation concerning pluripotency and reprogramming.

Next we examined the E8.5 embryos generated from ESCs in tetraploid rescue experiments for the presence of PGC-like cells by staining for AP (Lawson et al., 1999). We found a striking difference in embryos generated from *Blimp1*^−/−^ rESCs in which we saw no AP-positive cluster at the base of the allantois (6/8), except for fewer than 6 AP-positive cells near the base of the allantois in 2 embryos, which did not seem to be migrating appropriately like authentic PGCs (Figures 3D and 3E). By contrast, we observed normal clusters of PGCs in control embryos (Figure 3C). We previously demonstrated that AP-positive cells in Blimp1 mutant embryos lack all the attributes of authentic PGCs; instead, they have some characteristics of neighboring somatic cells with the expression of certain *Hox* genes, absence of PGC markers, and lack of expression of key pluripotency genes such as *Sox2,* and they undergo apoptosis after a lack of proliferation (Ohinata et al., 2005). Similar results were obtained with an independently derived *Blimp1*^−/−^ rESC line of mixed genetic background (Figure 3F). These findings mirror the phenotype of similar AP-positive aberrant cells observed in *Blimp1*^−/−^ embryos, obtained by heterozygous crosses, at the same stage.

These results show that loss of Blimp1 does not prevent derivation of epiSCs or their reversion to rESCs. The combined data also show that *Blimp1*^−/−^ rESCs are similar to control rESCs by transcriptome analysis as they both contribute to adult chimeras, except that the mutant cells cannot undergo specification into PGCs and be transmitted through the germline; they may also not contribute to some somatic tissues where Blimp1 is required later in development as shown previously (Robertson et al., 2007). Because rESCs can be derived from Blimp1-null epiSCs, this provides evidence indicating that epigenetic reprogramming inherent to the reversion process does not involve obligatory dedifferentiation of PGCs, and importantly, unequivocally excludes an obligatory transition through Blimp1-positive PGC-like state.

### Conclusion

Blimp1 is obligatory for PGC specification but it does not appear to be required for the derivation or the maintenance of pluripotent ESCs or of *Blimp1*^−/−^ epiSCs. Importantly, *Blimp1*^−/−^ epiSCs can undergo appropriate epigenetic reprogramming and reversion to rESCs in response to LIF-STAT3 signaling. This is a very stringent test for whether or not Blimp1 is essential, since reversion entails X reactivation and DNA demethylation; this occurred in the presence of LIF-serum and did not require 2i culture conditions. Reprogramming of Blimp1-null epiSCs to *Blimp1*^−/−^ rESCs conclusively excludes their transition through a Blimp1-positive PGC-like state. Because PGC specification and establishment of the germline is impossible without Blimp1, it is reasonable to conclude that reversion of epiSCs to rESCs does not transit through an equivalent PGC-like state.

**Figure 4:**
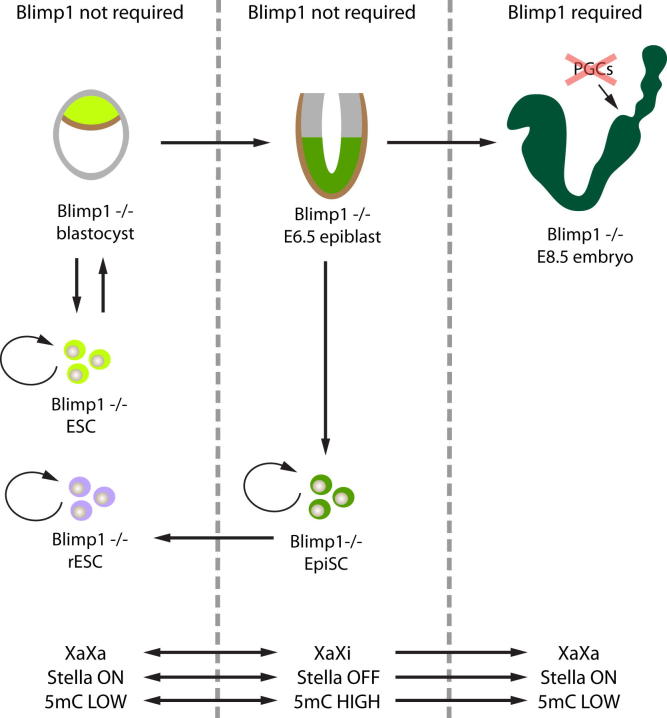
Representation of Blimp1 Requirement in Pluripotency, Reprogramming, and Germ Cells Blimp1 is not essential for the derivation and the maintenance of pluripotent ESCs or epiSCs. Reprogramming of epiSCs to rESCs, which is accompanied by epigenetic changes such as DNA demethylation and X reactivation that are also detected in the early germline, can also occur in the absence of Blimp1. By contrast, Blimp1 is critical for PGC specification and epigenetic reprogramming in early germ cells, which is mechanistically unrelated to the reprogramming of epiSCs to rESCs that does not entail an obligatory route through a PGC-like state.

Blimp1 and Prdm14 together are critical for epigenetic reprogramming during specification of PGCs and early germ cells. Reprogramming in early germ cells shares some key features with the reversion of epiSCs to rESCs. However, our study shows that reprogramming of epiSCs to rESCs, and possibly of somatic cells to iPSCs, does not require Blimp1, although PRDM14 appears to be essential for the maintenance of human and potentially mouse ESCs but not the mouse epiSCs (Chia et al., 2010; A.G. and M.A.S., unpublished observation). By contrast, Blimp1 is not required for the derivation and maintenance of mouse ESCs or epiSCs. Indeed, Blimp1 is rapidly downregulated during reprogramming of normal PGCs to pluripotent embryonic germ cells (EGCs), suggesting that Blimp1 is critical for the maintenance of unipotent germ cells, but it may restrict reversion to a pluripotent state because EGCs are equivalent to ESCs (Durcova-Hills et al., 2008; Leitch et al., 2010). Thus, PGC specification from epiblasts and epiSCs on the one hand and reversion of epiSCs to rESCs on the other (Figure 4) serves as a good model to gain novel insights on diverse mechanisms underlying epigenetic reprogramming in different contexts.

## Experimental Procedures

### Embryos

Timed natural matings were used for all experiments, where noon of the day when the vaginal plugs of mated females were identified was scored as E0.5. MEFs were obtained from E13.5 fetuses from the MF1 strain. Animal studies were authorized by a UK Home Office Project License and carried out in a Home Office-designated facility.

### Derivation of Mouse ESCs from *Blimp1*^−/−^ Blastocysts

ESC lines were derived essentially as described previously (Nichols et al., 2009). For single-cell deposition experiments, single-cell suspensions from each trypsinized ICM were dispersed in one well of a 48-well plate containing HS-27 feeders (available from ATCC). 2i/LIF medium comprises N2B27 basal medium (Stem Cells Inc.) supplemented with 1 μM PD0325901, 3 μM CHIR99021 (Signaling Technologies, University of Dundee), and mouse LIF (10 μg/ml, University of Cambridge Department of Biochemistry).

### Production of E6.5 Epiblast in Tetraploid Host Blastocysts

Two-cell stage embryos (E1.5) from F1 (C57BL/6 × CBA) matings were collected by flushing oviducts; these were subjected to electrofusion to create tetraploid (4N) host blastocysts (Nagy et al., 2003). Typically 15–20 *Blimp1*^−/−^ ESCs were injected into tetraploid host blastocyts, which were transferred to E2.5 pseudopregnant recipients, and examined at E6.5.

### *Blimp1*^−/−^ EpiSC Derivation and Culture

EpiSCs were derived from E6.5 epiblasts by culturing on MEFs in N2B27 medium containing human activin A (20 ng/ml; Peprotech), bFGF (12 ng/ml; Invitrogen), and KSR (20%; Invitrogen) (Bao et al., 2009). The cells were passaged every 3 days as described previously. When the colonies increased in size, they were dissociated with collagenase IV (1 mg/ml; Invitrogen) until the establishment of epiSCs after about 10 passages.

### Reversion of Mouse *Blimp1*^−/−^ EpiSC to rESC Lines

*Blimp1*^−/−^ epiSCs (∼passage 20) were treated with collagenase for 8 min at room temperature and transferred to a dish with feeders and standard ESC medium (1,000 U/ml LIF, 20% FCS in DMEM/F12 medium). After 12 to 30 days, colonies of 100 to 200 μm diameter were detected, within which we detected clusters of cells with a different morphology. These cultures were dissociated by trypsin into single-cell suspension and passaged on feeders and standard ESC medium. ESC-like cell were detected several days later and established as mutant or control rESCs.

### Alkaline Phosphatase Staining

AP staining of epiSCs and rESCs was carried out according to manufacturer's instructions (Roche). In brief, the cells were fixed in 4% paraformaldehyde for 10 min and stained overnight at room temperature. AP staining of PGCs was performed as described previously (Lawson et al., 1999).

### Quantitative Real-Time PCR

Total RNA was extracted with the RNeasy Mini Kit (QIAGEN) with DNase treatment. 500 ng of total RNA were used for cDNA synthesis with Superscript III (Invitrogen) and random hexamer primers (Invitrogen). qRT-PCR reactions were set up with Sybr Green JumpStart Taq ReadyMix (Sigma) and 1 μM of forward and reverse gene-specific primers (see Table S3 for primer sequences). Amplification was performed with an ABI Prism 7000 Sequence Detection System (Applied Biosystems) at 95°C for 10 min, 40 cycles of 95°C for 15 s and 60°C for 1 min, followed by a melting curve. Mean threshold cycles were determined from two technical replicates by the comparative Ct method and expression levels were normalized to GAPDH.

### Microarray Analysis

Total RNA was prepared with the RNeasy Mini Kit (QIAGEN) with DNaseI treatment. Eluted RNA concentration was determined by spectrophotometry. After RNA quality control with the Bioanalyzer, the samples were processed and hybridized to Illumina Mouse WG-6 v2.0 Expression BeadChips by Cambridge Genomic Services, who also performed data quality control. Raw data were loaded into lumi (Du et al., 2008) and then divided into subsets to be analyzed. The data were transformed by variance stabilization (VST) (Lin et al., 2008) and normalized with quantile normalization. Comparisons were performed by limma (Smyth, 2004) and the results corrected by false discovery rate (FDR). Microarray data are presented as a correlation heatmap that depicts the correlation between samples.

### Immunostaining

Cells were briefly washed with PBS and fixed in 4% paraformaldehyde in PBS for 15 min at room temperature. Cells were permeabilized for 30 min with 1% BSA and 0.1% Triton X-100 in PBS. Antibody staining was carried out in the same buffer at 4°C overnight. The slides were subsequently washed three times in PBS, 1% BSA, and 0.1% Triton X-100 (5 min each wash), incubated with secondary antibody for 1 hr at room temperature in the dark, and washed once for 5 min in PBS, 1% BSA, and 0.1% Triton X-100 and twice for 5 min in PBS. The slides were then mounted in Vectashield with DAPI (Vector Laboratories) and imaged with a BioRad Radiance 2100 confocal microscope. Primary antibodies used were mouse monoclonal Oct4 (BD Biosciences, 1:200), rabbit polyclonal Nanog (Abcam, 1:200). All secondary antibodies used were Alexa Fluor highly crossed adsorbed (Molecular Probes).

### Detection of PGC-like Cells

We injected *Blimp1*^−/−^ rESCs derived from *Blimp1*^−/−^ epiSCs into tetraploid host blastocysts and transferred them to E2.5 recipients. E8.5 embryos were isolated and PGC-like cells were counted after AP staining. The reprogrammed rESCs derived from epiSCs with Oct4-ΔPE-GFP reporter were used as a control.

## Accession Numbers

The microarray data are available in the Gene Expression Omnibus (GEO) database (http://www.ncbi.nlm.nih.gov/gds) under the accession number GSE35983.

## References

[bib1] Bao S., Tang F., Li X., Hayashi K., Gillich A., Lao K., Surani M.A. (2009). Epigenetic reversion of post-implantation epiblast to pluripotent embryonic stem cells. Nature.

[bib2] Brons I.G., Smithers L.E., Trotter M.W., Rugg-Gunn P., Sun B., Chuva de Sousa Lopes S.M., Howlett S.K., Clarkson A., Ahrlund-Richter L., Pedersen R.A., Vallier L. (2007). Derivation of pluripotent epiblast stem cells from mammalian embryos. Nature.

[bib3] Chia N.Y., Chan Y.S., Feng B., Lu X., Orlov Y.L., Moreau D., Kumar P., Yang L., Jiang J., Lau M.S. (2010). A genome-wide RNAi screen reveals determinants of human embryonic stem cell identity. Nature.

[bib4] Chu L.F., Surani M.A., Jaenisch R., Zwaka T.P. (2011). Blimp1 expression predicts embryonic stem cell development in vitro. Curr. Biol..

[bib5] Du P., Kibbe W.A., Lin S.M. (2008). lumi: a pipeline for processing Illumina microarray. Bioinformatics.

[bib6] Durcova-Hills G., Tang F., Doody G., Tooze R., Surani M.A. (2008). Reprogramming primordial germ cells into pluripotent stem cells. PLoS ONE.

[bib7] Greber B., Wu G., Bernemann C., Joo J.Y., Han D.W., Ko K., Tapia N., Sabour D., Sterneckert J., Tesar P., Schöler H.R. (2010). Conserved and divergent roles of FGF signaling in mouse epiblast stem cells and human embryonic stem cells. Cell Stem Cell.

[bib8] Hajkova P., Ancelin K., Waldmann T., Lacoste N., Lange U.C., Cesari F., Lee C., Almouzni G., Schneider R., Surani M.A. (2008). Chromatin dynamics during epigenetic reprogramming in the mouse germ line. Nature.

[bib9] Han D.W., Greber B., Wu G., Tapia N., Araúzo-Bravo M.J., Ko K., Bernemann C., Stehling M., Schöler H.R. (2011). Direct reprogramming of fibroblasts into epiblast stem cells. Nat. Cell Biol..

[bib10] Hanna J., Markoulaki S., Mitalipova M., Cheng A.W., Cassady J.P., Staerk J., Carey B.W., Lengner C.J., Foreman R., Love J. (2009). Metastable pluripotent states in NOD-mouse-derived ESCs. Cell Stem Cell.

[bib11] Hayashi K., Surani M.A. (2009). Self-renewing epiblast stem cells exhibit continual delineation of germ cells with epigenetic reprogramming in vitro. Development.

[bib12] Lawson K.A., Dunn N.R., Roelen B.A., Zeinstra L.M., Davis A.M., Wright C.V., Korving J.P., Hogan B.L. (1999). Bmp4 is required for the generation of primordial germ cells in the mouse embryo. Genes Dev..

[bib13] Leitch H.G., Blair K., Mansfield W., Ayetey H., Humphreys P., Nichols J., Surani M.A., Smith A. (2010). Embryonic germ cells from mice and rats exhibit properties consistent with a generic pluripotent ground state. Development.

[bib14] Lin S.M., Du P., Huber W., Kibbe W.A. (2008). Model-based variance-stabilizing transformation for Illumina microarray data. Nucleic Acids Res..

[bib15] Ma Z., Swigut T., Valouev A., Rada-Iglesias A., Wysocka J. (2011). Sequence-specific regulator Prdm14 safeguards mouse ESCs from entering extraembryonic endoderm fates. Nat. Struct. Mol. Biol..

[bib16] Nagamatsu G., Kosaka T., Kawasumi M., Kinoshita T., Takubo K., Akiyama H., Sudo T., Kobayashi T., Oya M., Suda T. (2011). A germ cell-specific gene, Prmt5, works in somatic cell reprogramming. J. Biol. Chem..

[bib17] Nagy A., Gertsenstein M., Vintersten K., Behringer R. (2003). Manipulating the Mouse Embryo: a Laboratory Manual.

[bib18] Nichols J., Smith A. (2011). The origin and identity of embryonic stem cells. Development.

[bib19] Nichols J., Silva J., Roode M., Smith A. (2009). Suppression of Erk signalling promotes ground state pluripotency in the mouse embryo. Development.

[bib20] Ohinata Y., Payer B., O'Carroll D., Ancelin K., Ono Y., Sano M., Barton S.C., Obukhanych T., Nussenzweig M., Tarakhovsky A. (2005). Blimp1 is a critical determinant of the germ cell lineage in mice. Nature.

[bib21] Robertson E.J., Charatsi I., Joyner C.J., Koonce C.H., Morgan M., Islam A., Paterson C., Lejsek E., Arnold S.J., Kallies A. (2007). Blimp1 regulates development of the posterior forelimb, caudal pharyngeal arches, heart and sensory vibrissae in mice. Development.

[bib22] Smyth G.K. (2004). Linear models and empirical Bayes methods for assessing differential expression in microarray experiments. Stat. Appl. Genet. Mol. Biol..

[bib23] Surani M.A., Hayashi K., Hajkova P. (2007). Genetic and epigenetic regulators of pluripotency. Cell.

[bib24] Tesar P.J., Chenoweth J.G., Brook F.A., Davies T.J., Evans E.P., Mack D.L., Gardner R.L., McKay R.D. (2007). New cell lines from mouse epiblast share defining features with human embryonic stem cells. Nature.

[bib25] Vincent S.D., Dunn N.R., Sciammas R., Shapiro-Shalef M., Davis M.M., Calame K., Bikoff E.K., Robertson E.J. (2005). The zinc finger transcriptional repressor *Blimp1/Prdm1* is dispensable for early axis formation but is required for specification of primordial germ cells in the mouse. Development.

[bib26] Yamaji M., Seki Y., Kurimoto K., Yabuta Y., Yuasa M., Shigeta M., Yamanaka K., Ohinata Y., Saitou M. (2008). Critical function of Prdm14 for the establishment of the germ cell lineage in mice. Nat. Genet..

[bib27] Yang J., van Oosten A.L., Theunissen T.W., Guo G., Silva J.C., Smith A. (2010). Stat3 activation is limiting for reprogramming to ground state pluripotency. Cell Stem Cell.

[bib28] Ying Q.L., Wray J., Nichols J., Batlle-Morera L., Doble B., Woodgett J., Cohen P., Smith A. (2008). The ground state of embryonic stem cell self-renewal. Nature.

[bib29] Zwaka T.P., Thomson J.A. (2005). A germ cell origin of embryonic stem cells?. Development.

