# Supplementary material for: The Germ Cell Determinant Blimp1 Is Not Required for Derivation of Pluripotent Stem Cells
Source: Cell Stem Cell. 2012 Jul 6;11-20(1):110–7. doi: 10.1016/j.stem.2012.02.023 (PMC3391686; doi:10.1016/j.stem.2012.02.023)
Supplement: Document S1. Figures S1 and S2 and Tables S1–S3 [file mmc1.pdf]

Cell Stem Cell, Volume *11*

Supplemental Information

The Germ Cell Determinant Blimp1 Is Not Required for Derivation of Pluripotent Stem Cells

Siqin Bao, Harry G. Leitch, Astrid Gillich, Jennifer Nichols, Fuchou Tang, Shinseog Kim, Caroline Lee, Thomas Zwaka, Xihe Li, and M. Azim Surani

INVENTORY OF SUPPLEMENTAL INFORMATION

Figure S1 (related to Figure 2)

Figure S2 (related to Figure 3)

Table S1 (related to Figure 1)

Table S2 (related to Figure 1)

Table S3 (related to Figure 2)

A

Epiblast	EpiSC	Genotype	rESC
BL2	√	-/-	√
BL3	√	+/-	√
BL4	√	+/-	ND
BL5	√	+/-	ND
2BL1	√	+/+	ND
2BL2	x	x	x
2BL3	x	x	x
2BL4	√	+/-	ND
2BL5	√	+/+	√
2BL6	√	-/-	√
2BL7	√	+/-	√
2BL8	√	+/-	ND

B

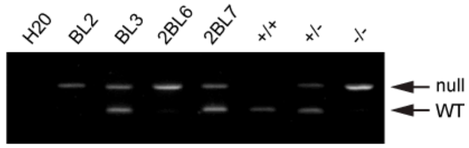

C

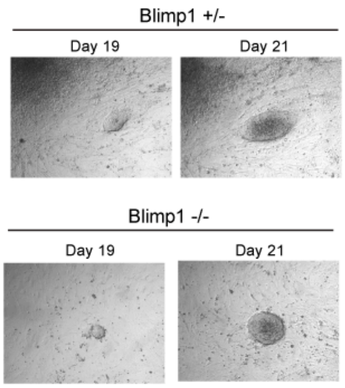

D

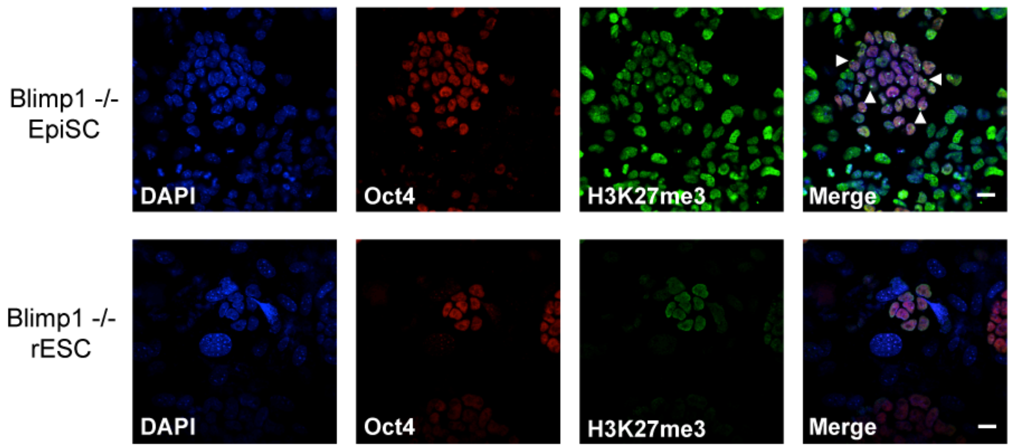

E

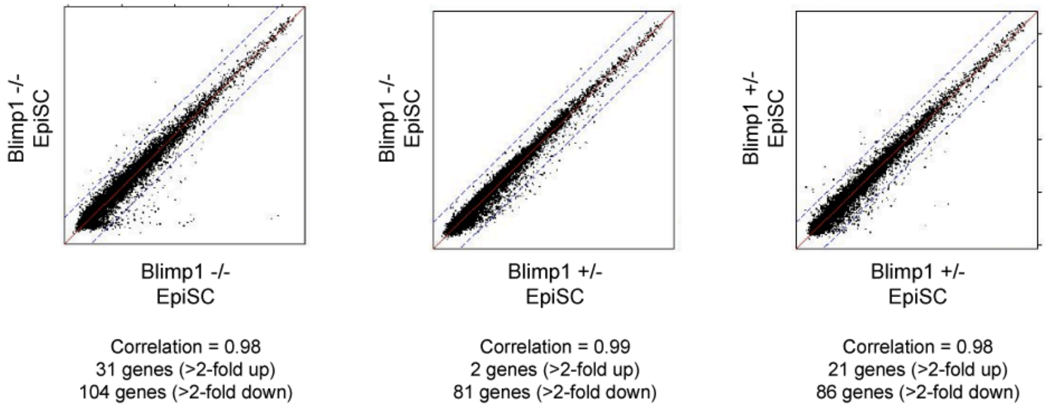

Figure S1. Characterisation of Blimp1 -/- EpiSC and their reversion to rESC.
(A) Table of EpiSC derivation from E6.5 epiblasts obtained from Blimp1 heterozygous intercrosses. 2 null, 2 heterozygous and 1 wildtype EpiSC were successfully reverted to rESC. ND = not done. (B) Representative genotyping results for 4 of the lines shown in (A). WT= wildtype band. (C) Representative images of emerging rESC on day 19 and 21 of reversion. (D) Immunostaining of Blimp1 -/- EpiSC and rESC for Oct4 and H3K27me3. H3K27me3 nuclear foci can be observed in Oct4-positive cells in EpiSC cultures (examples denoted by arrowheads) but not rESC derived from them. DNA counterstained with DAPI. Oct4-negative cells are feeders. Scale bars: 20 μm. (E) Scatter plot analysis of microarray data comparing Blimp1 null and heterozygous epiSC lines.

A

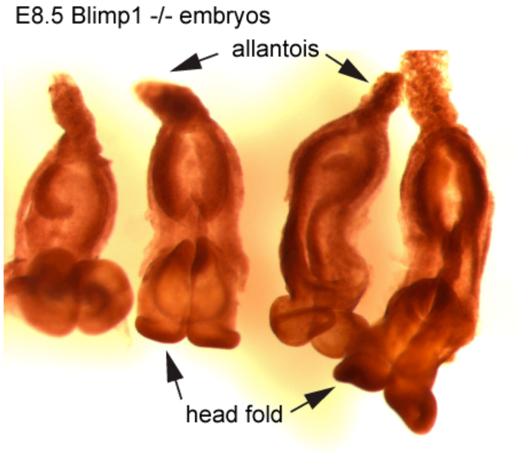

B

	No. of injected 4N blastocysts	No. of implantation sites	No. of E8.0 - 8.5 embryos
Blimp1 -/- rESC	47	29 (62%)	7 (24%)
Wild type rESC	35	23 (66%)	6 (26%)

Figure S2. Blimp1 null rESC can generate overtly normal E8.5 embryos in tetraploid rescue experiments. (A) E8.5 embryos obtained following injection of Blimp1 null rESC into tetraploid blastocysts. Stained with alkaline phosphatase. (B) Summary of a tetraploid injection experiment comparing Blimp1 null rESC with wildtype control rESC.

Table S1. Summary of an independent ES cell derivation experiment from a Blimp1+/- mating.

Embryo	ESC line (Yes/No)?	Blimp1 genotype
1	Yes	+/+
2	Yes	+/+
3	Yes	+/+
4	Yes	+/-
5	Yes	+/-
6	Yes	+/+
7	Yes	+/-
8	Yes	+/+
**9**	**Yes**	**-/-**
10	Yes	+/+

Table S2. Chimera generation from Blimp1-/- ESC

Blimp1 -/- ESC clone	No. blastocysts injected	No. born	No. chimaeras	Germline transmission?
1	25	8	6	No
2	23	5	4	No

Table S3. Sequences of qPCR primers

**qPCR Primers**		
Gapdh	CATGGCCTTCCGTGTTCCT	GCGGCACGTCAGATCCA
Oct4 (Pou5f1)	TGGATCCTCGAACCTGGCTA	CCCTCCGCAGAACTCGTATG
Nanog	AATGCTGCTCCGCTCCATAA	TAAAATGCGCATGGCTTTCC
Klf2	TAAAGGCGCATCTGCGTACA	CGCACAAGTGGCACTGAAAG
Fgf5	AAACTCCATGCAAGTGCCAAAT	TCTCGGCCTGTCTTTTCAGTTC
Klf4	GCACACCTGCGAACTCACAC	GTTTGCGGTAGTGCCTGGTC
Foxa2	TCTCCGTGTCAGGAGCACAA	AGGCAGGTGCTCCCTTTAGC
